# Meconium microbiome and its relation to neonatal growth and head circumference catch-up in preterm infants

**DOI:** 10.1371/journal.pone.0238632

**Published:** 2020-09-21

**Authors:** Ana Carolina Terrazzan Nutricionist, Renato S. Procianoy, Luiz Fernando Wurdig Roesch, Andrea Lúcia Corso, Priscila Thiago Dobbler, Rita C. Silveira

**Affiliations:** 1 Postgraduate Program in Child and Adolescent Health–PPGSCA, Federal University of Rio Grande do Sul–UFRGS, Porto Alegre, Brazil; 2 Neonatal Intensive Care Unit Hospital de Clínicas de Porto Alegre, RS, Porto Alegre, Brazil; 3 Interdisciplinary Center for Biotechnology Research–CIP-Biotec Federal University of Pampa, São Gabriel, Rio Grande do Sul, Brazil; Oswaldo Cruz Foundation, BRAZIL

## Abstract

The purpose was identify an association between meconium microbiome, extra-uterine growth restriction, and head circumference catch-up. Materials and methods: Prospective study with preterm infants born <33 weeks gestational age (GA), admitted at Neonatal Unit and attending the Follow-Up Preterm Program of a tertiary hospital. Excluded out born infants; presence of congenital malformations or genetic syndromes; congenital infections; HIV-positive mothers; and newborns whose parents or legal guardians did not authorize participation. Approved by the institution's ethics committee. Conducted 16S rRNA sequencing using PGM Ion Torrent meconium samples for microbiota analysis. Results: Included 63 newborns, GA 30±2.3 weeks, mean weight 1375.80±462.6 grams, 68.3% adequate weight for GA at birth. *Polynucleobacter* (p = 0.0163), Gp1 (p = 0.018), and *Prevotella* (p = 0.038) appeared in greater abundance in meconium of preterm infants with adequate birth weight for GA. Thirty (47.6%) children reached head circumference catch-up before 6 months CA and 33 (52.4%) after 6 months CA. *Salmonella* (p<0.001), *Flavobacterium* (p = 0.026), and *Burkholderia* (p = 0.026) were found to be more abundant in meconium in the group of newborns who achieved catch-up prior to 6th month CA. Conclusion: Meconium microbiome abundance was related to adequacy of weight for GA. Meconium microbiome differs between children who achieve head circumference catch-up by the 6th month of corrected age or after this period.

## Introduction

The balance between the host and intestinal microbes is protective to health [1–3]. Gut microbiota is essential for suitable nutrient absorption, energy storage, and immune response, and it’s also responsible for multiple metabolic tasks, including production of essential vitamins, fermentation and breakdown of oligosaccharides and production of short-chain fatty acids and gases [4, 5]. However, for the microbiota to perform such tasks, the host must maintain a favorable gut environment.

The mechanisms by which microbiota formation occurs via placenta and amniotic fluid are still not fully elucidated. Some studies support the hypothesis that fetal intestinal microbiome is derived from the swallowing of amniotic fluid containing bacteria [6, 7]. The mechanism related to this hypothesis is that maternal bacteria might translocate through maternal bloodstream, achieving other organs and systems, reaching amniotic fluid also [8] Yet, more studies are needed in order to better elucidate mechanisms involved in microbiota formation via placenta and amniotic fluid [9, 10].

There is evidence of a gut-brain axis, linking gut microbiota and the development of nervous system function. The maintenance of this bidirectional communication between central and enteric nervous system evolves endocrine, immune and neuronal pathways and it’s essential for neurological development and brain growth [11, 12].

For many reasons preterm infants are also high-risk infants for impaired growth, nutrition and neurodevelopment; and the possible early dysbiosis might interfere on microbiota metabolic capacity, and consequently alter nutrient absorption, influencing growth and neurodevelopment [1, 13].

A better understanding of microbiome variation may allow the early detection of a subpopulation of preterm infants at higher risk for growth and developmental impairment during follow-up. Thus, we aimed to identify and describe the composition of the microbiota of the first meconium of preterm infants. We also aimed to verify if there was an association between microbiota composition with restricted extra-uterine growth and with head circumference catch-up after discharge, both important growth variables that may influence the neurodevelopmental outcomes.

## Material and methods

The study was approved by the Institutional Ethics Committee of Hospital de Clinicas de Porto Alegre and Brazilian review board. All mother or legal guardian had provided written informed consent. This was a prospective cohort study including preterm infants gestational age <33 weeks, born and admitted at the Neonatal Unit and attending the Follow-Up Preterm Program of a tertiary hospital in Porto Alegre, RS. Infants born in another hospital, presence of congenital malformations or genetic syndromes, congenital infections, and HIV+ mother were exclusion criteria. Data collection started following Institution Ethics Committee approval (140009 –n°1.388.950). Clinical data and sample characterization were prospectively recorded and associated to meconium microbiome sequencing data bank. Maternal variables studied were: maternal age, mode of delivery, maternal antibiotics, presence of urinary tract infections (urine culture test positive and clinical signs), or clinical chorioamnionitis (maternal fever, uterine hypertonia, malodorous or purulent amniotic fluid, maternal leukocytosis or fetal tachycardia), preeclampsia, and gestational diabetes. Preeclampsia was defined as presence of hypertension (blood pressure > 140/90 mmHg after 20 weeks of gestation with significant proteinuria). For gestational diabetes, fasting was ≥ 92g/dL or glycemia of ≥153 g/dL following oral glucose tolerance test, with onset during pregnancy. Neonatal variables: gender, birth weight, gestational age (determined by the best obstetrical estimate, including first trimester ultrasound and/or last menstrual period date, confirmed by pediatric physical examination immediately after birth), being appropriate-for-gestational-age (AGA), small-for-gestational-age (SGA: below the 10th percentile according to reference curve), intrauterine growth restriction (below 3rd percentile). We also looked at hospitalization data to verify periintraventricular leukomalacia, necrotizing enterocolitis, early and late sepsis, hospitalization after discharge, and use of anticonvulsant.

Following NICU discharge, patients were referred to the Follow-Up Program. According to the routine of the institution, all children have monthly appointments up to 6 months of corrected age. Routine also includes anthropometric measurement (weight, length, head circumference). For this study, we evaluated head circumference at 2, 4, and 6 months corrected age in order to identify those patients for whom catch-up head circumference was achieved before or after 6 months corrected age. Catch-up was defined as a ≥ 0.67 z-score variation between two consecutive z-scores [14]. Fenton Growth Calculator for Preterm Infants (2013) [15] was used to generate birth data z-scores, as well as to determine adequacy of weight for gestational age; and WHO Anthro, 3.2.2 version (2011) was used for z-scores from follow-up period. Both software take into account gender and age, with age being corrected for preterm infants. Standardized equipment for measuring the infants was used by a trained researcher (ACT). Weight was measured using a digital scale, accurate to within 5g (ELP, 25BBA, Balmak®), with the infant wearing no clothes. Length was measured to the nearest centimeter in horizontal position using a length board accurate to 0.1 cm, with the infant lying down. Head circumference was measured using a non-stretch tape, accurate to 0.1 cm, placed on the broadest part of the forehead above eyebrows, above the ears, and around the most prominent part of the back of the head.

Feeding practices, regarding type of milk the infants were receiving (mother’s milk, infant formula, or cow’s milk) were evaluated, from hospital discharge up to six months corrected age.

### Meconium collection samples

After the mother or legal guardian had provided written informed consent, the first meconium passed by the infant was collected from diaper in sterile conditions, immediately stored at -80°C in a cryogenic storage Dewar, and transported to a laboratory where microbial DNA extraction and microbial community composition analysis was performed. This collection occurs mandatorily before the newborn receives any enteral feeding, as some studies suggest differences in microbial colonization between breastfed infants and formula-fed infants [16].

### Microbial DNA extraction, amplification, and sequencing

Microbial DNA was isolated from 180 mg of each meconium sample using the QIAamp Fast DNA Stool Mini Kit (Qiagen, Valencia, CA, USA), in accordance with manufacturer instructions. DNA quality was verified by spectrophotometry in a NanoVue™ system (GE Healthcare, Chicago, IL, USA). All DNA samples were stored at -80°C until use. V4 region of 16S rRNA gene was amplified and sequenced using ION PGM™ Ion Torrent (Thermo Fisher Scientific, Waltham, MA, USA), with primers 515F and 806R. Multiple samples were amplified by polymerase chain reaction (PCR) using barcoded primers linked to adapter “A” sequence (5′-CCATCTCATCCCTGCGTGTCTCCGACTCAG-3′) and “P1” sequence (5′-CCTCTCTATGGGCAGTCGGTGAT-3′) to obtain a primer sequence composed for the A-barcode-806R and P1-515F adapter and primers. PCR reaction final volume was 25 μL. Each mix consisted of 2U Platinum® Taq DNA High Fidelity Polymerase (Invitrogen, Carlsbad, CA, USA), 4 μL 10X High Fidelity PCR Buffer, 2 mM MgSO4, 0.2 mM dNTPs, 0.1 μM of both primers described above, 25 μg UltraPure BSA (Invi-trogen, Carlsbad, CA, USA), and approximately 50 ng of template DNA.

PCR conditions used were: 95°C for 5 min, 35 cycles at 94°C for 45 s, 56°C for 45 s, and 72°C for 1 min, followed by 72°C for 10 min. Resulting PCR products were purified with Agencourt® AMPure® XP Reagent (Beckman Coulter, La Brea, CA, USA) and quantified using the Qubit Fluorometer kit (Invitrogen, Carlsbad, CA, USA), following manufacturer recommendations.

Finally, reactions were combined in equimolar concentrations to create a mixture composed of amplified fragments of 16S gene from each sample. This composite sample was used for library preparation with OneTouch™ 2 Ion system using the ION™ PGM Template 400 OT2 kit (Thermo Fisher Scientific, Waltham, MA, USA). Sequencing was performed using commercially available ION PGM™ Sequencing 400 kit on an ION PGM™ System, using an Ion 318™ Chip v2, with a maximum of 40 samples per microchip.

### Sequence processing for analysis

Fastq files exported from ION PGM™ system were analyzed following recommendations from Brazilian Microbiome Project (BMP) [17], using the BMP Operating System [18]. Briefly, an Operational Taxonomic Unit (OTU) table was compiled using UPARSE pipeline [19] wherein sequences were truncated at 200 base pairs and quality filtered using a maximum expected error cutoff of 0.5. Sequences were clustered into OTUs using a 97% similarity cutoff, and chimeric sequences were removed. Taxonomic classification was performed in QIIME software environment [20], based on UCLUST method, against Greengenes 13.5 database [21], with a confidence limit of 80%. Sampling effort was estimated using Good’s coverage formula [22]. For downstream analysis, the data set was filtered by removing Chloroplast/Cyanobacteria sequences and only OTUs with more than 5 sequence reads were kept before rarefying all samples to 5379 sequences each [23].

Functional prediction for the gut microbiome was performed using PICRUSt 24]. For that, the raw 16S rRNA dataset was prepared following the instructions of Langille et al. (2013) [24]. After quality filtering and trimming, OTUs were picked against the Greengenes [21] database.

### Statistical analyses

Data obtained were stored in a database constructed for this specific purpose, using Excel software. Afterwards, data were processed and analyzed using PASW (SPSS) software, 18.0 version (Statistical Package for Social Sciences). Results are expressed as mean ± Standard Deviation (SD), minimum and maximum values, or median and interquartile (p25-p75). Differences between medians were analyzed with Mann-Whitney test. Between-groups differences were analyzed by T test, Qui Square, and ANOVA when more than two groups were analyzed.

Microbiome database was imported into R (R Development Core Team, 2008) to assess structural differences in the microbial community and detect possible confounders; a compositional dissimilarity matrix was generated based on the Bray-curtis distances between samples using the phyloseq package [25]. The matrix was used in a nonparametric Multivariate Analysis of Variance (PERMANOVA) with the Adonis function available in the vegan package [26]. To estimate alpha diversity, microbial dominance and Shannon diversity index were calculated and plotted using the "phyloseq" package [25]. Alpha diversity measurements were tested for normality with Shapiro-Wilk test and variables were compared by Kruskal-Wallis rank sum test. Differential abundance analysis was performed with DESEq2 [27]. The p-values were adjusted for multiple comparisons using the FDR method.

For the functional prediction of the gut microbiota, functions were categorized by the third KEGG Pathway Hierarchy Level and hypothesis testing was performed with two-sided White’s non-parametric t-test. Hypothesis testing and plotting were done using STAMP [28] Only features with a difference in proportion of 0.1 (Effect size > 0.1) were considered as active.

## Results

Eighty-seven samples were collected. Eleven were excluded for not being sterile, six did not have enough material for analysis, and in seven it was not possible to determine microbial DNA. In total, for this study we analyzed 63 meconium samples of preterm infants, of whom 30 (47.6%) were boys, with mean gestational age of 30±2.3 weeks. Mean weight, length, and head circumference at birth were 1375.80±462.6 grams, 38.0±4.0 centimeters, and 27±2.7 centimeters, respectively. Mean maternal age was 25.95±6.5 years, and 45 (71.4%) infants were delivered by C-section. Prevalence of preeclampsia, gestational diabetes, and urinary tract infection was 16(25.4%), 7 (11.1%), and 7 (11.1%), respectively. At discharge, mean gestational age was 38±3 weeks and mean weight was 2573.05±292.18 grams.

Forty-nine (68.3%) were AGA, and of these 57.14% (n = 36) were also discharged AGA. Thirteen (20.63%) were born AGA and were SGA at discharge. Twelve (19.4%) were born SGA and were discharged also SGA. Only two (3.17%) of those born SGA were LGA at discharge (this group was excluded from data analysis, because of its limited size). The growth pattern was significantly higher among the AGA neonates. Regarding use of breast milk or formula during the hospital stay, no difference was found according to adequacy of weight for gestational age at birth and discharge. (Table 1).

**Table 1:** Clinical characteristics of preterm infants according to adequacy of weight for gestational age at birth and discharge.

Variables	AGAbirth-AGAdischarge (n = 36)	AGAbirth-SGAdischarge (n = 13)	SGAbirth-AGAdischarge (n = 12)	SGAbirth-LGAdischarge (n = 2)	*p* value
Male**	16 (44.4%)	7 (53.85%)	6 (50.%)	1 (50%)	0.944
Maternal Age*(years)	25.92±6.69	25.62±6.13	26.58±6.62	27.5±10	0.973
C-section**	15 (41.7%)	2 (15.4%)	1 (8.3%)	0	0.062
Preeclampsia**	4 (11.1%) ^a^	4 (30.8%)^a.b^	6 (50%)^b^	2 (100%)^b^	0.003
GDM**	5 (13.9%)	1 (7.7%)	1 (8.3%)	0	0.855
UTI**	5 (13.9%)	1 (7.75)	1 (8.3%)	0	0.855
GA at birth* (weeks)	30.11±2.35	29.85±2.44	29.58±2.74	31.5±0.7	0.744
BW* (kg)	1.500±0.507 ^a^	1.3800±0.506 ^a.b^	1.000±0 ^b^	1.000±0 ^a.b^	0.010
BW z-score***	0.16 (-1.42–2.46) ^a^	-0.28 (-1.11–1.51)^a.c^	-1.65 (-2.08–-1.35) ^b^	-1.44 (-1.55–-1.34)^b.c^	<0.001
L at birth* (cm)	40.18±3.28 ^a^	38±3.69	34.5±5.1 ^b^	38±1.41	0.001
BL z-score ***	0.20 (-2.0–1.69) ^a^	-0.53 (-1.60–-0.67) ^b^	-1.83 (-3.42–-0.12) ^c^	-1.40 (-1.45–-1.35) ^a.b.c^	<0.001
CP at birth* (cm)	27.94±2.54 ^a^	27.38±2.3 ^a.b^	24.92±2.9 ^b^	25.3±0.49 ^a.b^	0.008
CP at birth z-score ***	0.13 (-1.66–2.05) ^a^	-0.14 (-1.48–1.35) ^a^	-1.67 (-2.40–-0.53) ^b^	-1.24 (-1.57–-0.92)^a.b^	<0.001
Length of hospitalization*** (days)	47.4(14–114)	63.3 (29–122)	72.8 (25–137)	48 (25–71)	0.104
GA discharge* (weeks)	36.8±2.24 ^a^	38.9±2.95^a.b^	39.9±3.86 ^b^	38.3±3.9 ^a.b^	0.008
Weight at discharge* (kg)	2.63±0.572	2.49±0.335	2.440±0.489	2.777±0.682	0.625
Type of milk at discharge**					
EBM	5 (13.9%)	2 (15.4%)	1 (8.3%)	1 (50%)	0.176
BM+formul	19 (52.8%)	6 (46.2%)	10 (83.3%)	1(50%)	
Formula	12 (33.3%)	5 (38.5%)	1(8,3%)	0	

*Mean ± SD;

**Absolut frequency (%);

***Mean (Min-Max); AGA: Appropriate-for-Gestational-Age; SGA: Small-for-gestational-age BW: Birth weight; L: Length; CP: Head circumference; GA: Gestational Age; GDM: gestational diabetes mellitus; UTI: Urinary Tract Infection; EBM: Exclusive Breast Milk; BM; Breast Milk

**Fig 1:**
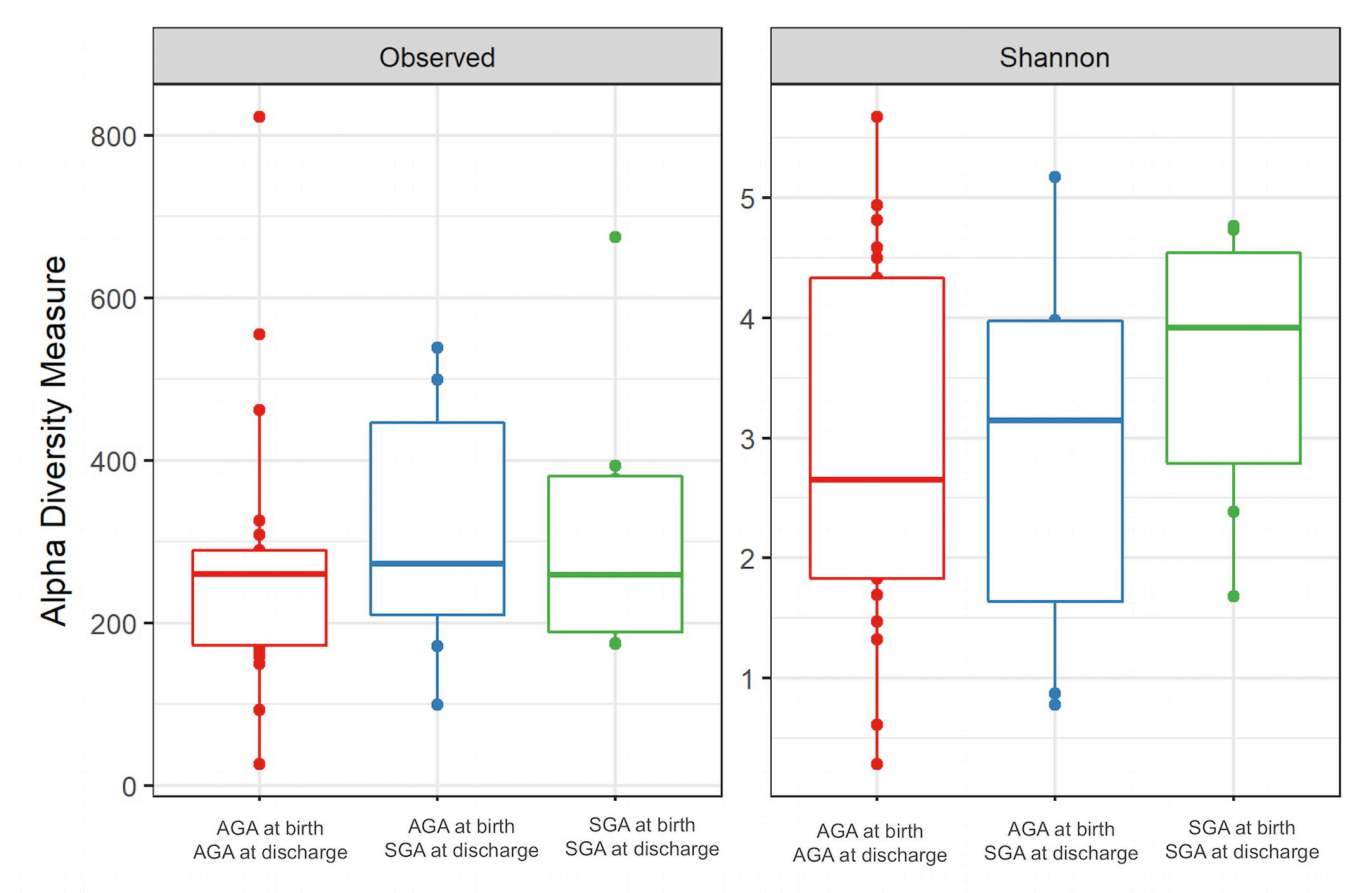
Alpha diversity measurements of meconium microbial communities from preterm infants comparing weight adequacy at birth and at discharge. The left panel presents the number of observed Operational Taxonomic Unities (OTUs) (p-value = 0.745). The right panel presents the Shannon microbial index of diversity (p-value = 0.127). Boxes span the first to third quartiles; the horizontal line inside the boxes represents the median. Whiskers extending vertically from the boxes indicate variability outside the upper and lower quartiles, and single circles indicate outliers. AGA: adequate for gestational age; SGA: small for gestational age. (The group SGA-LGA was excluded from data analysis, because of its limited size).

In total, we identified 5,309 different OTUs across all samples, of these, 16 OTUs had mean abundance higher than 1%. Microbial composition was similar when compared according to weight at birth and at discharge. Alpha diversity measurements between groups AGA-AGA vs. AGA-SGA vs. SGA-SGA were similar (Observed OTUs, p-value = 0.745) and Shannon Diversity Index, p-value = 0.127 (Fig 1).

**Fig 2:**
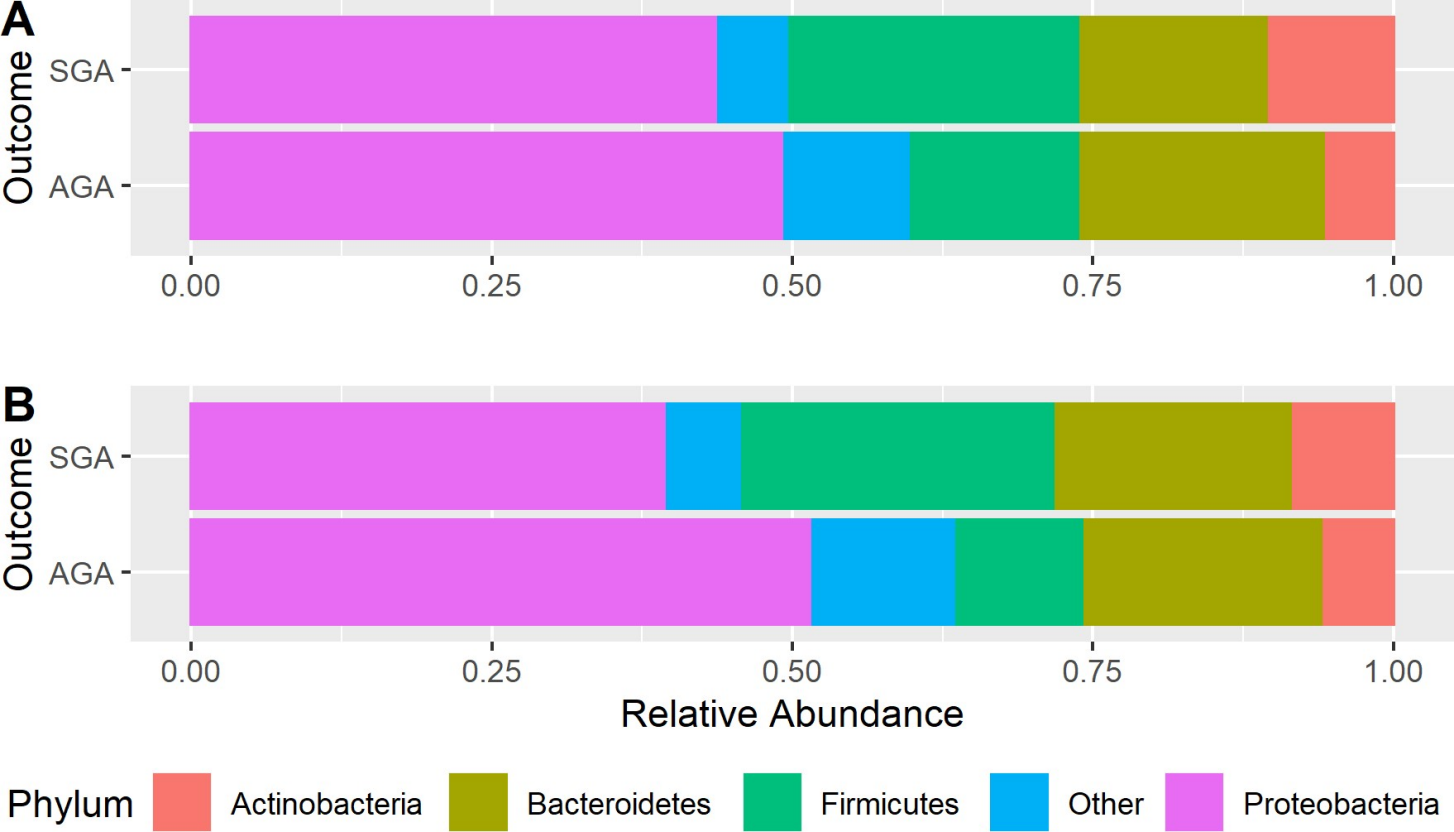
Relative phyla abundance of the gut microbiota according with weigh adequacy. Each stacked bar represents the mean relative abundance of weight adequacy group at birth (A) and at moment of discharge (B).

The overall microbial composition at phylum level according to weight adeqacy at birth is presented in Fig 2A, and at discharge in Fig 2B. Four phyla were found to be dominant across the samples irrespective of weight adequacy at birth or delivery. They were *Proteobacteria*, *Bacteroidetes*, *Firmicutes*, and *Actinobacteria*. On average, infants in the SGA group at birth or discharge had higher *Firmicutes* while those in the AGA group had higher *Proteobacteria* then their couterparts.

**Fig 3:**
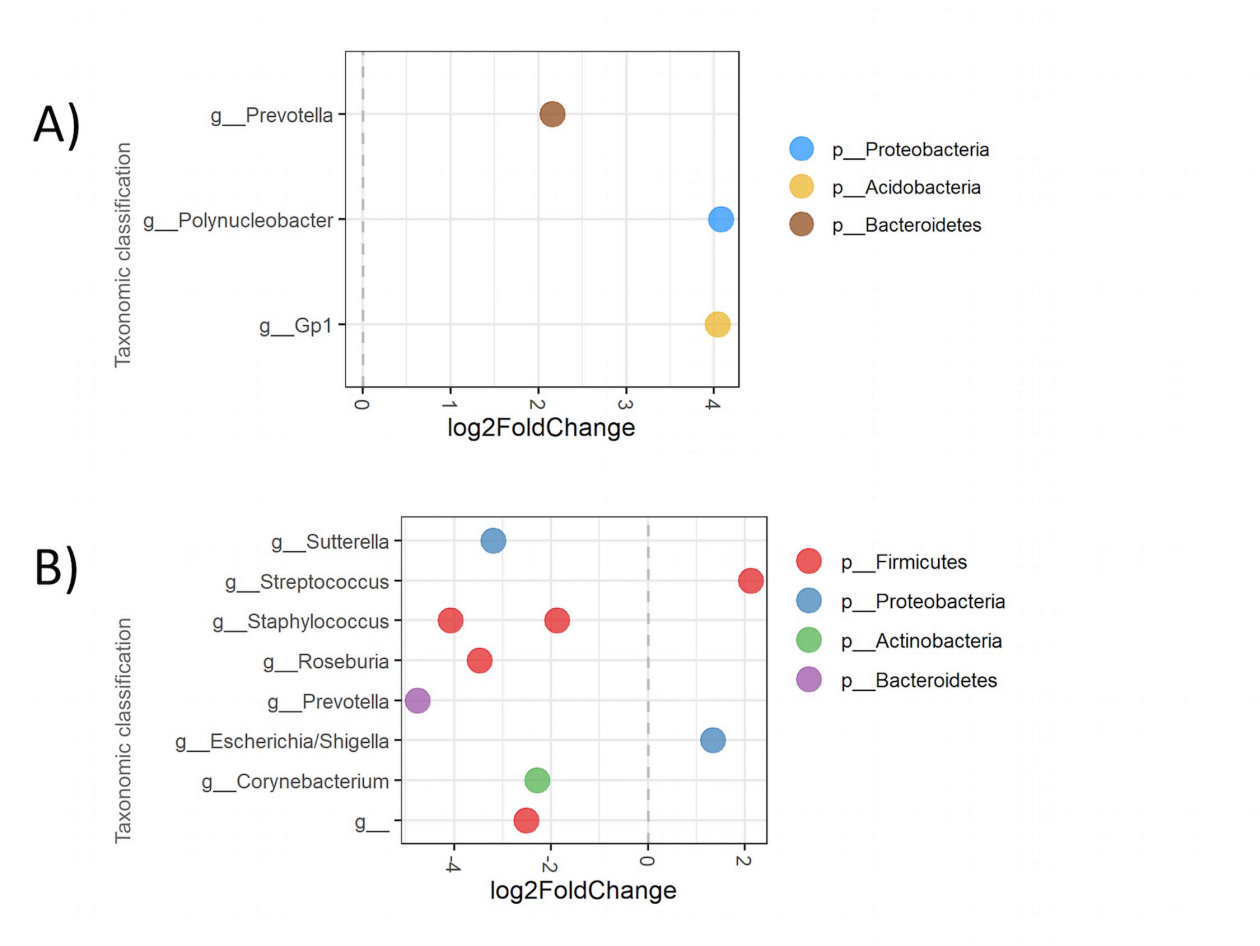
Differential abundance analysis according to weight adequacy. Each dot represents an individual OTU, organized by their Genus. **(A)** Differential abundance analysis according to weight adequacy at birth: *Polynucleobacter (p =* 0.0163), *Gp1 (p = 0*.*018) and Prevotella (p = 0*.*038)* were more abundant in meconium of preterm born AGA. **(B)** Differential abundance analysis according to weight adequacy for gestational age at discharge: *Escherichia fergusoni (p = 0*.*014)* and *Streptococcus dentisani (p = 0*.*43)* were more abundant in meconium of preterm AGA at discharge; *Prevotella copri (p = 0*.*002)*, *Roseburia inulinivorans (p = 0*.*003)*, *Staphylococcus* sp. (p = 0,003), *Staphylococcus capitis* subsp. *Capitis (p = 0*.*004)*, *Sutterella stercoricanis (p = 0*.*014)*, *Corynebacterium tuberculostearicum (p = 0*.*033) and Ruminococcaceae (0*.*043)* were more abundant in meconium of preterm SGA at discharge.

When compared to the SGA at birth group, those born AGA had an increased abundance of OTUs belonging to genus *Polynucleobacter* (p = 0.0163), phylum *Proteobacteria*, *Gp1* (p = 0.018) phylum *Acidobacteria*, and *Prevotella* (p = 0.038) phylum *Bacteriodetes* (Fig 3A).

Between most abundant OTUs observed, when comparing preterm AGA or SGA at discharge, those OTUs belonging to *Escherichia fergusoni* (p = 0.014) and *Streptococcus dentisani* (p = 0.043) genus were more abundant in the AGA at discharge group, and this difference was statistically significant. By contrast, the SGA at discharge group presented increased abundance of *Prevotella copri* (p = 0.002), *Roseburia inulinivorans* (p = 0.003), *Staphylococcus sp*. (p = 0.003), *Staphylococcus capitis* subsp. *Capitis* (p = 0.004), *Sutterella stercoricanis* (p = 0.027), *Corynebacterium tuberculostearicum* (p = 0.033), and *Ruminococcaceae* (p = 0.043) (Fig 3B)

Regarding head circumference (HC) catch-up growth, 30 (47.6%) infants completed HC catch-up growth by the age of 6 months corrected age and 33 (52.4%) after 6 months of corrected age. Also, catch-up occurred independently of weight adequacy for gestational age at birth or at discharge. There were no statistically significant differences regarding clinic variables at birth, sepsis during NICU stay, use of anticonvulsant, and rehospitalizations after discharge. As expected, the group that completed HC catch-up growth by the age of 6 months corrected age had higher z-score and measures of weight and head circumference between 2 and 6 months of corrected age. There was a difference between groups only at 6 months of corrected age, with a higher number of infants receiving infant formula in those whose HC catch-up growth was completed by the 6th month of corrected age (Table 2).

**Table 2:** Clinical characteristics, growth and type of milk received according to catch-up before or after 6 months of corrected age.

Variables	*Catch up* <6m (n = 30)	*Catch up* >6m (n = 33)	P value
Male**	16 (53.3%)	14 (42.4%)	0.454
Maternal age(years)*	25.33±6.26	27±6.77	0.299
C-section**	12 (40%)	18 (60%)	0.093
Preeclampsia**	4 (13.3%)	12 (36.4%)	0.046
Gestational Diabetes**	4 (13.3%)	3 (9.1%)	0.700
Urinary tract infection**	4 (13.3%)	3 (9.1%)	0.700
Maternal antibiotics**	20(66.7%)	21(63.6%)	1.000
GA at birth (weeks)*	30.4±2.29	29.6±2.4	0.209
AGA at birth**	22(73.3%)	26(78.8%)	0.612
Weight at birth (kg)*	1.434 ±0.443	1.323±0.479	0.345
Z-score Weight at birth***	-0.33 (-2.08–1.25)	-0.31 (-1.87–2.46)	0.933
Length at birth(cm)*	38.7±3.84	38.2±4.32	0.654
z-score Length at birth ***	-0.34 (-3.4–1.5)	-0.42 (-3.04–1.69)	0.788
Head circunference at birth* (cm)	27.52 ±2.66	26.55±2.92	0.247
Z-score Head circunference at birth ***	-0.27 (-2.36–1.79)	-0.34 (-2.4–2)	0.785
NICU stay (days)***	49 (14–114)	61 (29–122)	0.137
Periventricular leukomalacia	2 (6.7%)	3 (9.1.%)	0.546
Necrotizing enterocolitis	4 (13.3%)	6(18.2%)	0.430
Early sepsis	0	1 (3%)	0.625
Late sepsis	2 (6.6%)	3 (9%)	0.423
Gestational age at discharge (weeks)*	37.4±2.3	38.4±3.4	0.174
Weight at discharge (kg)*	2.63±0.572	2.49±0.335	0.625
Weight z-score at discharge ***	-0.94 (-3.2–1.38)	-1.35 (-3.33–0.27)	0.104
AGA at discharge**	18(60%)	19 (57.6%)	0.845
Hospitalization after discharge **	4 (13.3%)	10 (30.3%)	0.106
Use of anticonvulsant **	5 (16.7%)	10 (30.3%)	0.204
Weight at 2 months CA (kg)	5.450±0.970	4.98±0.810	0.055
Weight Z-score at 2 months CA	0 (-3.82–2.30)	-0.55 (-2.64–2.12)	0.134
Head circumference at 2 months CA (cm)	39.44±1.78	38.43±1.70	0.084
Head circumference Z-score at 2 months CA	0.75 (-2.69–2.87)	0 (-3.51–2.44)	0.040
Weight at 4 months CA (kg)	7.130±1.00	6.240v1.13	0.008
Weight Z-score at 4 months CA	0.37 (-1.81–2.66)	-0.68 (-4.31–2.44)	0.012
Head circumference at 4 months CA (cm)	42.57±1.14	40±2.0	<0.001
Head circumference Z-score at 4 months CA	1.13(-0.54–3.23)	-0.27 (-3.63–2.81)	0.001
Weight at 6 months CA (kg)	7.80±1.21	7.0±1.15	0.021
Weight Z-score at 6 months CA	0.05(-4.75–2.55)	-0.72(-4.38–2)	0.050
Head circumference at 6 months CA (cm)	44.1±1.25	41.71±1.96	<0.001
Head circumference Z-score at 6 months CA	0.94 (-1.92–2.75)	-0.39 (-3.55–2.14)	<0.001
Type of milk			
Milk at discharge			
EBM	4 (13.3%)	5 (15.2%)	0.090
BM+Formula	18 (60%)	18 (54.4%)	
Formula	8 (26.7%)	10 (30.3%)	
Milk at 2 months CA			
EBM	4 (14.3%)	6 (17.9%)	0.0752
BM+Formula	9 (28.6%)	11 (33.3%)	
Formula	15(53.6%)	16 (48.4%)	
Cows milk	1 (3%)	0	
Milk at 4 months CA			
EBM	2 (6.6%)	5(14.8%)	0.404
BM+Formula	8 (27.3%)	11(33.3%)	
Formula	19(63.6%)	17(51.9%)	
Cows milk	1 (3%)	0	
Milk at 6 months CA			
EBM	2(6.9)^a.b^	3 (7.4%)^a.b^	0.038
BM+Formula	3 (10.3%)	12 (37%)	
Formula	21 (69.9%)	18(55.6%)^b^	
Cows milk	4 (13.8%)	0^b^	

Mean ± SD;

**Absolut frequency (%);

***Mean (Min-Max); CA: corrected age; AGA: Appropriate-for-Gestational-Age; SGA: Small-for-gestational-age BW: Birth weight; L: Length; CP: Head circumference; GA: Gestational Age;; GDM: gestational diabetes mellitus; UTI: Urinary Tract Infection; EBM: Exclusive Breast Milk; BM; Breast Milk

**Fig 4:**
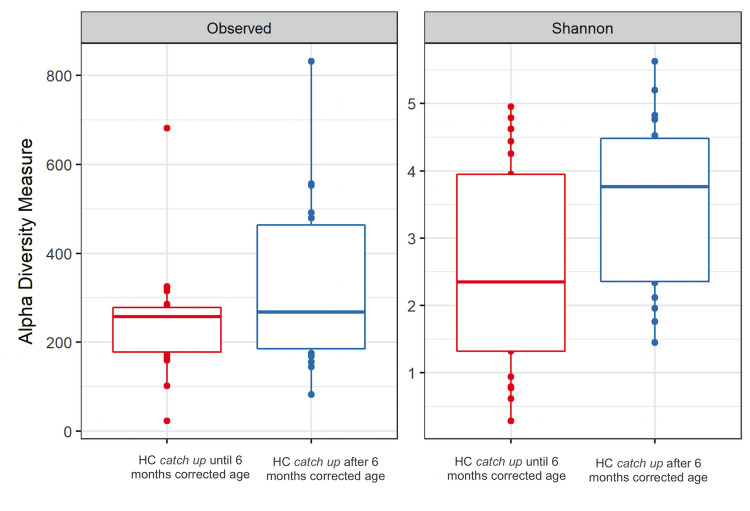
Alpha diversity measurements of meconium microbial communities from preterm infants comparing head circumference (HC) catch up until or after 6 months of corrected age. The left panel presents the number of observed Operational Taxonomic Unities (OTUs) (p-value = 0.225). The right panel presents the Shannon microbial index of diversity (p-value = 0.045). Boxes span the first to third quartiles; the horizontal line inside the boxes represents the median. Whiskers extending vertically from the boxes indicate variability outside the upper and lower quartiles, and single circles indicate outliers.

According to the PERMANOVA (Table 3) there was no statistically significant difference for microbial beta diversity between infants with early HC catch-up growth (up to 6 months) and late HC catch-up growth (after 6 months) (p = 0.093). However, after analyzing differences in microbial alpha diversity, Shannon Index was statistically significant (p = 0.045), indicating more microbial diversity in meconium from infants who had their HC catch-up growth later, after 6 months of corrected age (Fig 4). Pre-eclampsia was not associated to differences in the meconium microbiota (p-value = 0.64).

**Table 3:** Nonparametric Multivariate Analysis of Variance of bacterial community structure used for controlling confounding variables.

Variables	F Model	R^2^	p-value
Weight Adequacy	0.961	0.101	0.536
HC Catch-up	1.255	0.033	0.201
Preeclampsia	0.836	0.022	0.640

**Fig 5:**
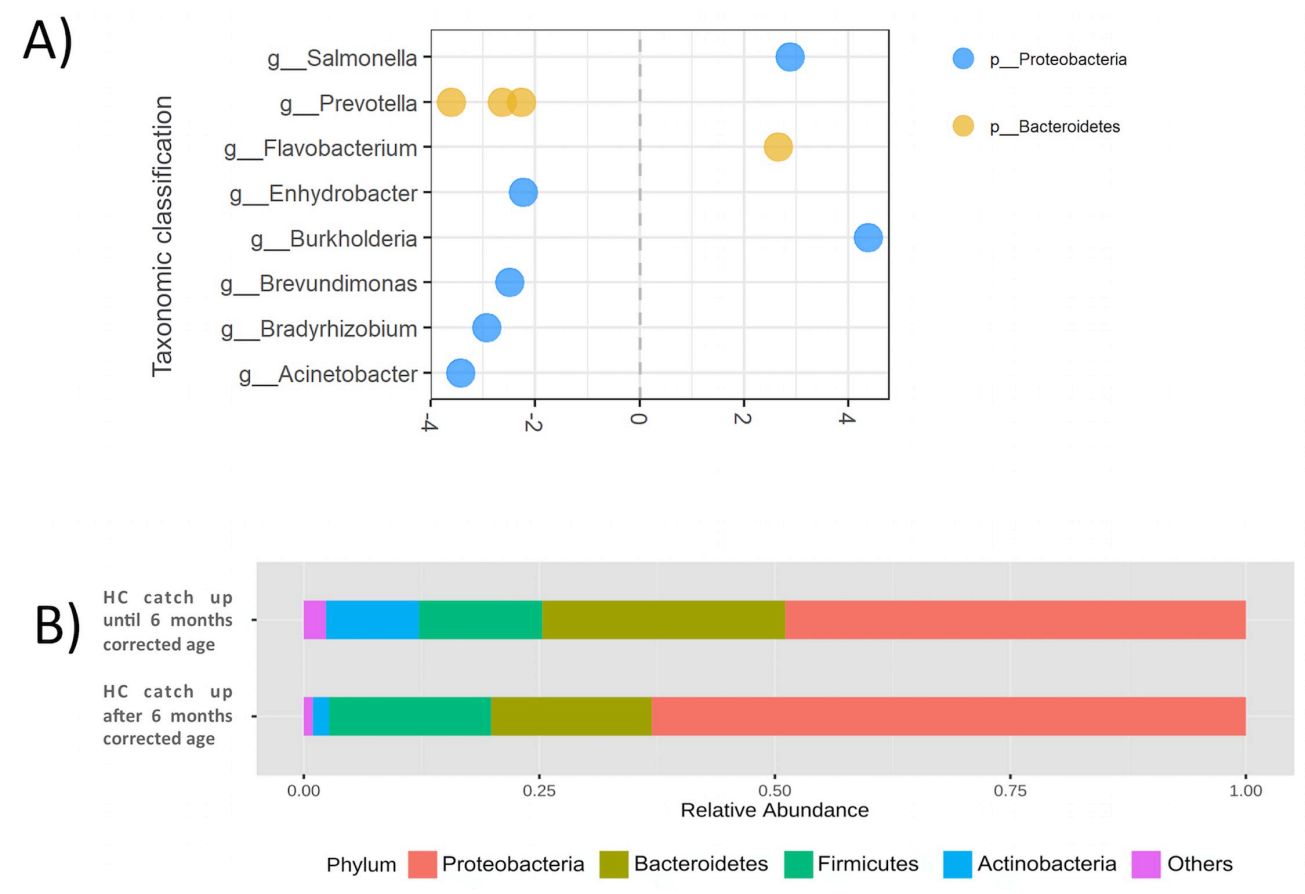
Differential abundance analysis according to head circumference catch up. Each dot represents an individual OTU, organized by their Genus. **(A)** Differential abundance analysis according to early or late HC catch up. Data plotted as log2 fold change; OTUs to the right of the zero line were more abundant in HC catch up until 6 months corrected age group, and OTUs to the left of the zero line were more abundant in HC catch up after 6 months corrected age group. **(B)** Difference for microbial composition between infants with early HC catch up growth (up to 6 months) and late HC catch up growth (after 6 months); HC: head circumference.

The overall microbial composition at phylum level within groups with the head circumference catch-up by 6 months and after 6 months is presented in Fig 5B. Four phyla were found to be dominant within the samples irrespective of the group. They were *Proteobacteria*, *Bacteroidetes*, *Firmicutes*, and *Actinobacteria*.

Differential abundance analysis showed increased abundance of *Bacterioidetes* and *Proteobacteria* phylum, with OTUs belonging to Salmonella (p<0.001), *Flavobacterium* (p = 0.026), and *Burkholderia* (p = 0.026) genus being the most abundant in meconium from infants who achieved HC catch-up growth by the 6th month of corrected age. *Prevotella* (p = 0.005), *Enhydrobacter* (p = 0.036), *Brevundinomonas* (p = 0.043), *Bradyhizobium* (p = 0.018), and *Acinetobacter* (p = 0.007) genus were more abundant in meconium of those infants who achieved HC catch-up growth after 6 months of corrected age (Fig 5A and 5B).

**Fig 6:**
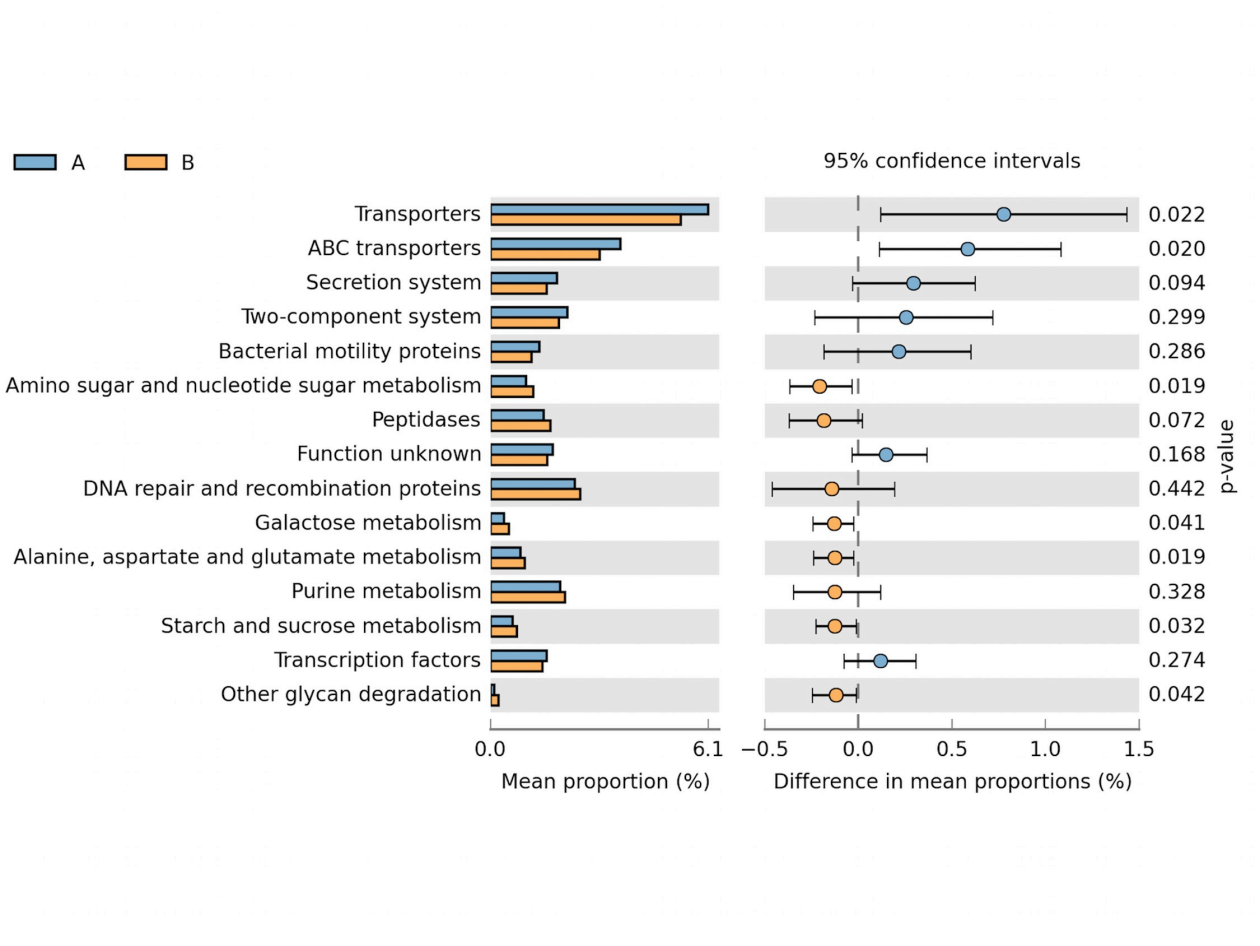
Microbial community functional prediction. Gut microbiota functional predictoin, using PICRUSt, of infants with early or late HC catch up. The bar plot respresents function mean proportion, and error bars represents the difference between the two groups. Coloring of the error bar is according with the group with the higher proportion of the respective function. Blue color (A) represents infants with HC catch up until 6 months, and Orange (B) represents those with HC catch up after 6 months of age.

In order to better understand the differences of the gut microbiota in relation with the time of HC catch up, we also explored the functional prediction of these communities, using PICRUSt [24]. Infants with HC catch up before the 6^th^ month of corrected age presented a microbiota with higher predicted genes relateted with transportation (Transporters and ABC transporters), while those with HC cacth up after 6 months had more genes related with sugar and amino acid metabolism (Fig 6).

When analysing functional gene prediction according with weight adequacy at birth or discharge, there were no significant differences, considering the treshold of effect size > 0.1 (S1 Fig).

## Discussion

Increased abundance of OTU belonging to *Prevotella*, *Polynucleobacter*, and *Gp1* genus in preterm infants born AGA was observed. Preterm AGA at discharge showed increased abundance of OTU belonging *to Escherichia fergusoni* and *Streptococcus dentisani* genus. We also found more abundance of OTUs *Salmonella*, *Flavobacterium*, and *Burkholderia* genus in the meconium of infants who achieved HC catch-up growth by the 6th month of corrected age. There are few studies with similar data; the great majority of studies consider the microbiome of full-term infants, and those that assess prematurity take into account only gestational age, without relating it to adequacy of weight for gestational age [29, 30].

Ardissone et al. (2014) [31] found several taxonomic families within *Firmicutes* phylum correlated to gestational age, including *Staphylococcus* genus, which were most abundant among preterms born at <33 gestational weeks. Jacquot et al. [32] found an association between gestational age less than 28 weeks and lower microbial diversity score at first week of life, where *Staphylococcus spp* genus was found in 67% of the patients. The authors also enlight that although it is clear that preterm infants can also present an important Staphylococcus colonization, these infants are at higher risk of late onset sepsis related to coagulase negative Staphylococcus during the first weeks of life [32].

Itani et al. (2017) [33] also described increased *Staphylococcus* abundance in feces from preterm infants less than 33 weeks of gestational age. Our data represent meconium microbiome, and we observed significantly increased *Staphylococcus* genus abundance in preterm infants who were SGA at discharge, with hospital discharge being equivalent to the term of gestational age. We hypothesize that besides gestational age, adequacy of weight for gestational age at birth is also related to microbial community structure. Also, although *Staphylococcus* colonization is a normal characteristic of healthy gut microbiota [34], we understand that a microbiota more abundant in *Staphylococcus* might interfere for nutrient absorption e metabolism, leading to a worse weigh gain during NICU stay, despite the efforts of nutrition therapy.

Nataro and Guerrant (2017) [35] suggest that *Prevotella* genus is associated to better growth, while *Streptococcus lutetiensis* and *Escherichia coli* are associated to growth failure, but they do not distinguish preterm from full-term infants. In our study, AGA at birth presented significant higher abundance of *Prevotella* genus, we believe this may reflect fetal period, once this microbe has been associated to improved glucose metabolism by promoting increased glycogen storage [36].

On the other hand, in contrast to Nataro and Guerrant (2017) [35] results, when we evaluate the adequacy of weight for gestational age at discharge, AGA preterms were the ones who presented increased *Escherichia fergusoni and Streptococcus dentisani* abundance in meconium, while SGA at discharge preterms presented increased *Prevotella copri* abundance in meconium. Through our results, we cannot infer about microbiota changes during the hospital stay, however, we have some hypothesis: a) Those infants with better growth (AGA at discharge) possibly had earlier contact with their parents and better evolution of dietary acceptance, both factors that can favor benefic changes in the microbiota. b) As we already mentioned, SGA infants at discharge also had abundant *Staphylococcus* in meconium and maybe during hospital stay this microbe was more resistant or had more impact host metabolism than *Prevotella copri*, influencing to the worse weigh gain. We understand that other external factors act together with the microbiome, being important influences in weight gain during hospital stay. Future studies, evaluating progressive changes in the microbiota, in association with dietary characteristics may answer this hypothesis.

Preterm infants miss an important phase of brain growth and maturation, which would occur during the last trimester of pregnancy [37]. During this phase the cortical gray matter is already matured, but some of the most important developing stages such as the increase in the complexity of connections, axons, glial cells, and oligodendrocytes in the withe matter, will be concluded as the 3rd trimester goes by [38, 39]. Therefore prematurity is associated with neurodevelopmental disability, with long term effects [3, 40, 41]. Catch down during hospital stay and during the first months of life are associated to increased risk of neurologic impairment in preterm infants, nevertheless the mechanisms that guarantee this association are not yet completely elucidated [6]. On the other hand, catch-up growth of head circumference in the first years of life is a protective factor for neurodevelopment, being associated to better cognitive and behavioral performance in early childhood [42, 43].

Taken together, neurological immaturity and a dysbiotic and immature gut, both associated with prematurity may disrupt the bidirectional communication between the nervous system and enteric cells, leading to altered signaling and neurological development, and also altered immune responses [3, 44, 45].

In the present study we were able to verify a higher microbial biodiversity in meconium from those children who had head circumference catch-up growth after 6 months of corrected age, with *Prevotella*, *Enhydrobacter*, *Brevundinomonas*, *Bradyhizobium*, and *Acinetobacter* being the most prevalent genus observed in the group. Moreover, in the group of infants whose head circumference catch-up growth was completed until 6 months of corrected age, *Salmonella*, *Flavobacterium*, and *Burkholderia* were most abundant. Community functional prediction suggests that the gut mictobiota of infants with head catch up until the 6^th^ month presented higher presence of transporter genes, including ABC transporters, while infants with head catch up after the 6^th^ month presented more genes predicted to be involved in the metabolism of complex carbohydrates, such as starch, and amino acids. This difference might influence energy intake from different sources and might influence growth.

Despite several studies aiming to explain the role of microbiome in the gut–brain axis, interactions between neurologic mechanisms and microbiome development in preterm infants are not well understood [38]. To our knowledge, this is the first study investigating meconium microbial composition and its association to head circumference catch-up growth in preterm infants. We suggest more studies should be conducted so that the pathways of this relationship may be better understood.

Guney Varal et al (2018) [46] conducted a study with preterm infants, using a prepared commercial symbiotic solution administered with enteral nutrition. Their results show a lower odd to lower head circumference growth in the study group. Wejryd et al (2018) [47] related supplementation with *L*. *reuteri* to better head circumference growth, also during hospital stay. Both studies corroborate the hypothesis that a favorable gut microbiota might enhance the chances of achieving better neurodevelopment/ growth via the beneficial effects on cytokines, nervous and immune system. However, a recent systematic review conducted by Hortensius et al (2019) [48] suggests that until the present, despite the positive results on head growth, there is no significant data regarding the effect of supplementation with probiotics on neurodevelopmental outcome was found. Therefore, it’s indeed necessary more follow up studies.

Experimental studies with germ-free mice have observed systemic inflammation and neuroinflammation in the offspring as well as impaired myelination and blood–brain barrier formation. These studies suggest a relationship between microbial colonization, immune system, and brain activity, as well as an essential role for microbiota in neural, structural, and functional development [45, 49]. Although animal model studies have already clearly elucidated the role of gut microbiota in childhood development programming, and there is a window of opportunity in which microbiota can affect physiological function of several systems, with long-term consequences, there have been only a limited number of studies with humans, specifically preterm newborns, that would enable complete understanding of processes involving microbiome and neurologic development [50].

Several factors such as infection, neurologic impairment, diet, and antibiotic use are crucial in ensuring growth. In our study the groups were similar for sepsis. However, post-discharge hospitalizations, anticonvulsant treatment, and milk feeding were different at 6 months of corrected age, which may directly interfere with growth, neurodevelopment, and microbial colonization. Thus, we cannot infer if meconium microbiota was the only determinant factor for head circumference catch-up growth. Yet, considering the intimate relationship between brain and gut [51], we suggest identifying microbiome variations associated and predisposing to accelerated head circumference catch-up growth as a relevant tool for clinical practice in the context of improving care and future health of preterm infants.

It is worth mentioning that food directly influences bacterial flora establishment, and human milk is a greater promoter of *Bifidobacteria* and *Lactobacillus* colonization when compared to formula based on cow’s milk [52]. Oligosaccharides (HMO) present in breast milk, which are complex glycans and not digestible by humans, are the main microbiome substrate, especially for *Bifidobacteria*, playing a fundamental role for beneficial bacterial community proliferation in children’s gut, due to both probiotic and prebiotic effects, highlighting the importance of promoting breastfeeding in the NICU environment [52–54].

It was a challenge to analyze the relationship between microbiome, born SGA or AGA, and head circumference catch-up growth, since there are so few studies and many unanswered questions. This study encountered limitations, such as the lack of microbiome data at discharge and follow up, which could give us more information regarding changes that occurred during hospital stay. We also understand the sample size as a limitation of this study; on the other hand, we emphasize the follow-up of preterm infants as strength.

## Conclusion

Meconium microbial abundance seems to be related to adequacy of weight for gestational age as well as to weight gain during neonatal period in low-birth-weight preterm infants. Also, abundance of meconium OTUs from infants who achieved early head circumference catch-up growth (defined in this study as up to the 6th month of corrected age) differs from those who had late head circumference catch-up growth (in this study, after 6 months of corrected age). Further studies following changes in microbial colonization, as well as its associations to diet patterns, in order to verify associations between microbiota and medium-term outcomes, may lead to new conduct definitions for clinical practice.

## Supporting information

S1 Fig Microbial community functional prediction. Infant’s gut microbiota functional predictoin, using PICRUSt regarding weight adequacy at birth (A) and at discharge (B). Here are all function predictions with an effect size > 0.1. The bar plot respresents function mean proportion, and error bars represents the difference between the two groups.
(TIFF)

S1 File Grants support. (DOCX)

## References

[1] Krajmalnik-Brown R, Ilhan ZE, Kang DW, DiBaise JK. Effects of Gut Microbes on Nutrient Absorption and Energy Regulation. 2012; 27:201–214 10.1177/0884533611436116 PMC3601187 22367888

[2] Robertson RC, Manges AR, Finlay BB, Prendergast AJ. The Human Microbiome and Child Growth–First 1000 Days and Beyond. Trends in Microbiology 2019; 27: 131–147 10.1016/j.tim.2018.09.008 30529020

[3] Lu J, Claud E. Connection between gut microbiome and brain development in preterm infants. Dev Psychobiol. 2019; 61: 739–751 10.1002/dev.21806 30460694 PMC6728148

[4] Rowland I, Gibson G, Heinken A, Scott K, Swann J, Thiele I, Tuohy K. Gut microbiota functions: metabolism of nutrients and other food componentes. Eur J Nutr. 2018; 57:1–24 10.1007/s00394-017-1445-8 PMC5847071 28393285

[5] Turroni F, Milani C, Durant S, Lugli GA, Bernasconi S, Margolles A, et al The infant gut microbiome as a microbial organ influencing host well-being. Ital J Pediatr. 2020 46:16 10.1186/s13052-020-0781-0 32024556 PMC7003403

[6] Jiménez E, Marín ML, Martín R, Odriozola JM, Olivares M, Xaus J, Fernández L, e tal. Is meconium from healthy newborns actually sterile? Res. Microbiol. 2008; 159: 187–193. 10.1016/j.resmic.2007.12.007 18281199

[7] Mshvildadze M, Neu J, Shuster J, Theriaque D, Li N, Mai V. Intestinal Microbial Ecology in Premature Infants Assessed Using Non-Culture Based Techniques. J Pediatr. 2010; 156: 20–25. 10.1016/j.jpeds.2009.06.063 19783002 PMC3628625

[8] Collado Maria Carmen & Rautava Samuli & Aakko, Juhani & Isolauri, Erika & Salminen, Seppo. Human gut colonisation may be initiated in utero by distinct microbial communities in the placenta and amniotic fluid. Scientific Reports. 2016; 6: 23129 10.1038/srep23129 27001291 PMC4802384

[9] Jimenez E, Fernandez L, Marin ML, Martín R, Odriozola JM, Nueno-Palop C, et al Isolation of commensal bacteria from umbilical cord blood of healthy neonates born by cesarean section. Curr Microbiol. 2005; 51:270–274 10.1007/s00284-005-0020-3 16187156

[10] Perez-Muñoz ME, Arrieta M-C, Ramer-Tait AE, Walter J. A critical assessment of the “sterile womb” and “in utero colonization” hypotheses: implications for research on the pioneer infant microbiome. Microbiome. 2017; 5:48 10.1186/s40168-017-0268-4 28454555 PMC5410102

[11] Rogers GB, Keating DJ, Young RL, Wong M-L, Licinio J, Wesselingh S. From gut dysbiosis to altered brain function and mental illness: mechanisms and pathways. Molecular Psychiatry. 2016;21:738–748 10.1038/mp.2016.50 27090305 PMC4879184

[12] Niccolai E, Boem F, Russo E, Amedei A. The Gut–Brain Axis in the Neuropsychological Disease Model of Obesity: A Classical Movie Revised by the Emerging Director “Microbiome”. Nutrients. 2019; 11:156. 10.3390/nu11010156 PMC6356219 30642052

[13] Henderickx JGE, Zwittink RD, van Lingen RA, Knol J and Belzer C. The Preterm Gut Microbiota: An Inconspicuous Challenge in Nutritional Neonatal Care. Front. Cell. Infect. Microbiol. 2019; 9:85 10.3389/fcimb.2019.00085 31001489 PMC6454191

[14] Ong KK, Ahmed ML, Emmett PM, Preece MA, Dunger DB. Association be-tween postnatal catch-up growth and obesity in childhood: prospective cohort study. BMJ. 2000; 320:967–71. 10.1136/bmj.320.7240.967 10753147 PMC27335

[15] Fenton TR, Kim JH. A systematic review and meta-analysis to revise the Fen-ton growth chart for preterm infants. BMC Pediatr. 2013;13:59 10.1186/1471-2431-13-59 23601190 PMC3637477

[16] Fanaro S, Chierici R, Guerrini P, Vigi V. Intestinal microflora in early infancy: composition and development. Acta Paediatr Suppl 2003; 91:48–55. 10.1111/j.1651-2227.2003.tb00646.x 14599042

[17] Pylro VS, Roesch LF, Ortega JM, do Amaral AM, Tótola MR, Hirsch PR, et al Brazilian Microbiome Project Organization Committee. Brazilian Microbiome Project: revealing the unexplored microbial diversity—challenges and prospects.Microb Ecol. 2014; 67: 237 10.1007/s00248-013-0302-4 24173537

[18] Pylro VS, Morais DK, de Oliveira FS, Dos Santos FG, Lemos LN, Oliveira G, et al BMPOS: a Flexible and User-Friendly Tool Sets for Microbiome Studies. Microbial Ecology.2016; 72: 443–447. 10.1007/s00248-016-0785-x 27220974

[19] Edgar R.C. UPARSE: Highly accurate OTU sequences from microbial amplicon reads. Nature Methods. 2013; 10:996–8 10.1038/nmeth.2604 23955772

[20] Caporaso JG, Kuczynski J, Stombaugh J, Bittinger K, Bushman FD, Costello EK, et al QIIME allows analysis of high-throughput community sequencing data. Nat Methods. Nature Methods. 2010; 10.1038/nmeth.f.303 PMC3156573 20383131

[21] McDonald D, Price MN, Goodrich J, Nawrocki EP, DeSantis TZ, Probst A, et al An improved Greengenes taxonomy with explicit ranks for ecological and evolutionary analyses of bacteria and archaea. ISME J. 2012; 6: 610–618. 10.1038/ismej.2011.139 22134646 PMC3280142

[22] Good IJ. The population frequencies of species and the estimation of popula-tion parameters. Biometrika.1953; 40: 237±264.

[23] Lemos LN, Fulthorpe RR, Triplett EW, Roesch LFW. Rethinking microbial diversity analysis in the high throughput sequencing era. Journal of Microbiological Methods. 2011; 86:42–51 10.1016/j.mimet.2011.03.014 21457733

[24] Langille MGI, Zaneveld J, Caporaso JG, McDonald D, Knights D, Reyes J. et al Predictive functional profiling of microbial communities using 16S rRNA marker gene sequences. Nature Biotechnology. 2013;1–10 10.1038/nbt.2482 23975157 PMC3819121

[25] McMurdie PJ, Holmes S. phyloseq: an R package for reproducible interactive analysis and graphics of microbiome census data. PLoS ONE. 2013; 8: e61217 10.1371/journal.pone.0061217 23630581 PMC3632530

[26] Oksanen J, Blanchet G, Kindt R, Legendre P, O'Hara R, Simpson G, et al Vegan: Community Ecology Package. 2011.

[27] Love MI, Huber W, Anders S. Moderated estimation of fold change and dispersion for RNA-seq data with DESeq2. Genome Biology. 2014; 15:550 10.1186/s13059-014-0550-8 25516281 PMC4302049

[28] Parks DH, Tyson GW, Hugenholtz P, Beiko RG. STAMP: Statistical analysis of taxonomic and functional profiles. Bioinformatics. 2014; 30: 3123–3124. 10.1093/bioinformatics/btu494 25061070 PMC4609014

[29] La Rosa PS, Warner BB, Zhou Y, Weinstock GM, Sodergren E, Hall-Moore CM et al Patterned progression of bacterial populations in the premature infant gut. Proc Natl Acad Sci. 2014;111:12522–7. 10.1073/pnas.1409497111 25114261 PMC4151715

[30] Korpela K, Blakstad EW, Moltu SJ, Strømmen K, Nakstad B, Rønnestad AE, et al Intestinal microbiota development and gestational age in preterm neonates. Scientific Reports. 2018; 8: 2453 10.1038/s41598-018-20827-x 29410448 PMC5802739

[31] Ardissone AN, de la Cruz DM, Davis-Richardson AG, Rechcigl KT, Li N, Drew JC et al Meconium Microbiome Analysis Identifies Bacteria Correlated with Premature Birth. PLoS ONE. 2014; 9: e90784 10.1371/journal.pone.0090784 24614698 PMC3948723

[32] Jacquot A, Neveu D, Aujoulat F, Mercier G, Marchandin H, Jumas-Bilak E, et al Dynamics and Clinical Evolution of Bacterial Gut Microflora in Extremely Premature Patients. J Pediatr. 2011; 158:390–6 10.1016/j.jpeds.2010.09.007 20961563

[33] Itani T, Ayoub Moubareck C, Melki I, Rousseau C, Mangin I, Butel MJ, et al Establishment and de-velopment of the intestinal microbiota of preterm infants in a Lebanese ter-tiary hospital. Anaerobe. 2017; 43:4–1 10.1016/j.anaerobe.2016.11.001 27833033

[34] Rinninella E, Raoul P, Cintoni M, et al What is the Healthy Gut Microbiota Composition? A Changing Ecosystem across Age, Environment, Diet, and Diseases. Microorganisms. 2019;7:14 10.3390/microorganisms7010014 PMC6351938 30634578

[35] Nataro J; Guerrant R. Chronic consequences on human health induced by microbialpathogens: Growth faltering among children in developing countries. Vaccine. 2017 35: 6807–6812 10.1016/j.vaccine.2017.05.035 28549806

[36] Kovatcheva-Datchary P, Nilsson A, Akrami R, Lee YS, De Vadder F, Arora T. Dietary Fiber-Induced Improvement in Glucose Metabolism Is Associated with Increased Abundance of Prevotella. Clinical And Translational Report. 2015; 22: 971–982. 10.1016/j.cmet.2015.10.001 26552345

[37] Cai S., Zhang G., Zhang H. et al Normative linear and volumetric biometric measurements of fetal brain development in magnetic resonance imaging. Childs Nerv Syst. 2020 10.1007/s00381-020-04633-3 32468242

[38] Lu L; Claud EC. Intrauterine Inflammation, Epigenetics, and Microbiome In-fluences on Preterm Infant Health. Current Pathobiology Reports. 2018 6:15–21 10.1007/s40139-018-0159-9 29938128 PMC5978889

[39] Volpe JJ. The encephalopathy of prematurity—brain injury and impaired brain development inextricably intertwined. Semin Pediatr Neurol. 2009;16: 167–78 10.1016/j.spen.2009.09.005 19945651 PMC2799246

[40] Volpe JJ. Brain injury in premature infants: a complex amalgam of destructive and developmental disturbances. Lancet Neurol. 2009; 8: 110–124 10.1016/S1474-4422(08)70294-1 19081519 PMC2707149

[41] Cheong J.L.Y., Burnett A.C., Treyvaud K. et al Early environment and long-term outcomes of preterm infants. J Neural Transm. 2020; 127: 1–8 10.1007/s00702-019-02121-w 31863172

[42] Belfort MB, Rifas-Shiman SL, Sullivan T, Collins CT, McPhee AJ, Ryan P, et al Infant growth before and after term: effects on neurodevelopment in pre-term infants. Pediatrics 2011;128:e899–906. 10.1542/peds.2011-0282 21949135 PMC3182845

[43] Ramel SE, Demerath EW, Gray HL, Younge N, Boys C, Georgieff MK. The relationship of poor linear growth velocity with neonatal illness and two year neurodevelopment in preterm infants. Neonatology 2012;102:19–24 10.1159/000336127 22441508

[44] Bäckhed F, Roswall J, Peng Y, Feng Q, Jia H, Kovatcheva-Datchary P. Dynamics and Stabilization of the Human Gut Microbiome during the First Year of Life. Resource. 2015; 17:690–703 10.1016/j.chom.2015.04.004 25974306

[45] Lu J, Lu L, Yu Y, Cluette-Brown J, Martin CR, Claud EC. Effects of Intestinal Microbiota on Brain Development in Humanized Gnotobiotic Mice. Scientific Reports. 2018:1–16 10.1038/s41598-017-17765-5 29615691 PMC5882882

[46] Guney Varal I, Koksal N, Ozkan H, Bagci O, Dogan P. Potential use of multi-strain synbiotics for improving postnatal head circumference. Pak J Med Sci. 2018;34:1502–1506 10.12669/pjms.346.16107 30559812 PMC6290199

[47] Wejryd E., Marchini G., Frimmel V., Jonsson B. and Abrahamsson T. Probiotics promoted head growth in extremely low birthweight infants in a double‐blind placebo‐controlled trial. Acta Paediatr. 2019; 108: 62–69 10.1111/apa.14497 29999201

[48] Hortensius LM, van Elburg RM, Nijboer CH, Benders MJNL and de Theije CGM. Postnatal Nutrition to Improve Brain Development in the Preterm Infant: A Systematic Review From Bench to Bedside. Front. Physiol. 2019; 10:961 10.3389/fphys.2019.00961 31404162 PMC6677108

[49] Hoban AE, Stilling RM, Ryan FJ, Shanahan F, Dinan TG, Claesson MJ et al Regulation of prefrontal cortex myelination by the microbiota. Transl. Psychiatry. 2016; 6:e774 10.1038/tp.2016.42 27045844 PMC4872400

[50] Ruiz L, Moles L, Gueimonde M, Rodriguez JM. Perinatal Microbiomes' Influence on Preterm Birth and Preterms' Health: Influencing Factors and Modulation Strategies. J Pediatr Gastroenterol Nutr. 2016; 63:e193–e203 10.1097/MPG.0000000000001196 27019409

[51] DiBartolomeo ME, Claud EC. The Developing Microbiome of the Preterm Infant. Clin Ther. 2016 38:733–739 10.1016/j.clinthera.2016.02.003 26947798 PMC4851874

[52] Guaraldi F, Salvatori G. Effect of breast and formula feeding on gut microbiota shaping in newborns. Front Cell Infect Microbiol. 2012; 2:94 10.3389/fcimb.2012.00094 23087909 PMC3472256

[53] Petherick A. Development: Mother’s milk: A rich opportunity. Nature. 2010; 468:S5–S7 10.1038/468S5a 21179083

[54] Victora CG, Bahl R, Barros AJ, França GV, Horton S, Krasevec J, et al Lancet Breastfeeding Series Group. Breastfeeding in the 21st century: epidemiology, mechanisms, and lifelong effect. Lancet. 2016; 387: 475–490 10.1016/S0140-6736(15)01024-7 26869575

