# Supplementary figures and images for: Meconium microbiome and its relation to neonatal growth and head circumference catch-up in preterm infants
Source: PLoS One. 2020 Sep 21;15(9):e0238632. doi: 10.1371/journal.pone.0238632 (PMC7505439; doi:10.1371/journal.pone.0238632)

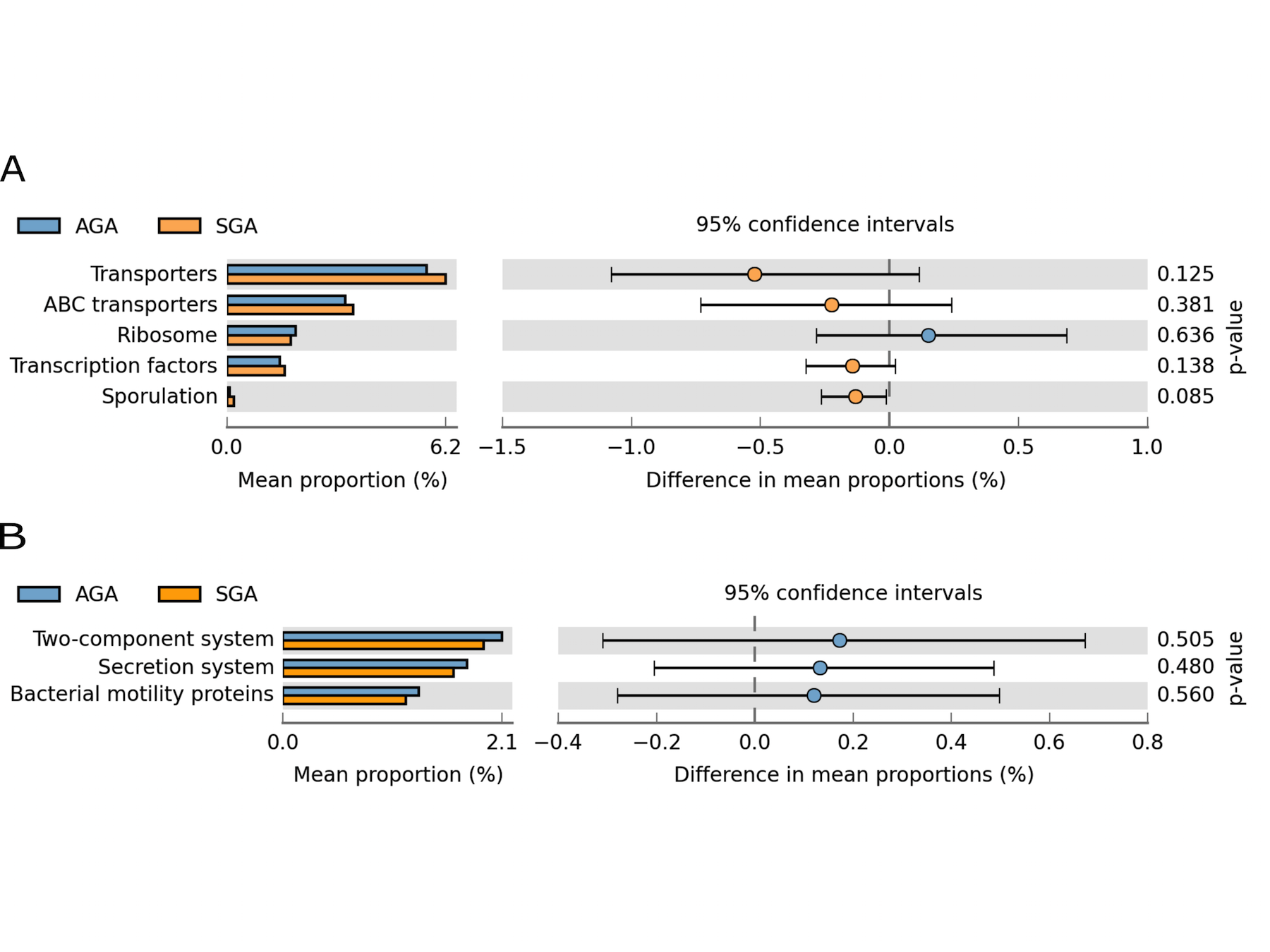

Supplement: S1 Fig — Infant’s gut microbiota functional predictoin, using PICRUSt regarding weight adequacy at birth (A) and at discharge (B). Here are all function predictions with an effect size > 0.1. The bar plot respresents function mean proportion, and error bars represents the difference between the two groups. (TIFF) [file pone.0238632.s001.tiff]
