# Supplementary material for: Meconium microbiome and its relation to neonatal growth and head circumference catch-up in preterm infants
Source: PLoS One. 2020 Sep 21;15(9):e0238632. doi: 10.1371/journal.pone.0238632 (PMC7505439; doi:10.1371/journal.pone.0238632)
Supplement: S1 File — (DOCX) [file pone.0238632.s002.docx]

This study was supported by a grant from Bill and Melinda Gates Foundation, CNPQ and DECIT / Health Ministry, Brazil, Grant number 401596/2013-3. This study was also financed in part by the Coordenação de Aperfeiçoamento de Pessoal de Nível Superior, Brazil (CAPES), Finance Code 001. The funders had no role in study design, data collection and analysis, decision to publish, or preparation of the manuscript.
